# Supplementary material for: Quantitative theory of magnetic properties of elemental praseodymium
Source: NPJ Comput Mater. 2025 Nov 3;11(1):326. doi: 10.1038/s41524-025-01803-2 (PMC12583131; doi:10.1038/s41524-025-01803-2)
Supplement: Supplementary file 1 — Supplementary information [file 41524_2025_1803_MOESM1_ESM.pdf]

Supplementary Information for "Quantitative theory of magnetic properties of elemental praseodymium".

Leonid V. Pourovskii[1,2*], Alena Vishina[3], Olle Eriksson[3,4], Mikhail I. Katsnelson[4,5]

[1*]CPHT, CNRS, École polytechnique, Institut Polytechnique de Paris, Palaiseau, Paris, 91120, France.
[2]Collège de France, Université PSL, 11 place Marcelin Berthelot, Paris, 75005, France.
[3]Department of Physics and Astronomy, Uppsala University, Box 516, Uppsala, 75120, Sweden.
[4]WISE-Wallenberg Inititative Materials Science, Uppsala University, Box 516, Uppsala, 75120, Sweden.
[5]Institute for Molecules and Materials, Radboud University, Heijendaalseweg 135, Nijmegen, 6525AJ, The Netherlands.

*Corresponding author(s). E-mail(s): leonid.poyurovskiy@polytechnique.edu;

S1 Interatomic exchange of hcp structures

In Fig. 1 we present the $\tilde{I}_{ij}$ parameters for hypothetical hcp Pr obtained in current work as well as the exchange parameters calculated in Ref. [1] for hcp Nd, and in Ref [2] for hcp Gd and hcp Tm. In Fig. 1 we divide $\tilde{I}_{ij}$ by $S(S+1)$ to account for the length of quantum spins, see Eqns. 7 and 8 of the main text. In the hcp structure, for all rare-earth elements analysed here, we observe a qualitatively similar behaviour with ferromagnetic nearest-neighbour interactions changing into oscillating values at larger distances, as reported previously for other lanthanides. Quantitatively, $\tilde{I}_{ij}/S(S+1)$ differ significantly among the calculated RE hcp metals, reflecting the sensitivity of the RE intersite exchange to changes in the lattice parameters.

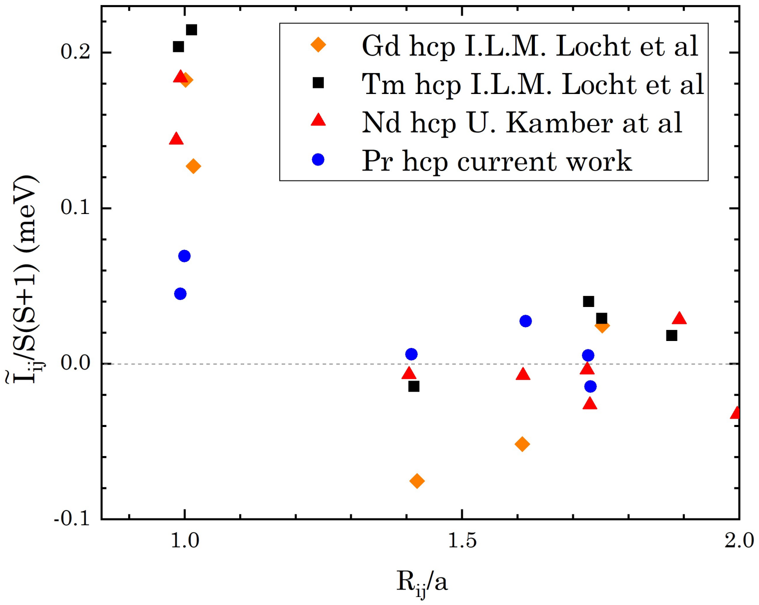

Supplementary Figure 1 Exchange interactions $\tilde{I}_{ij}/S(S+1)$ for the hcp structures of Pr (current work) compared to the values obtained for hcp Nd [1], hcp Gd [2], and hcp Tm [2].

S2 Interatomic exchange of the surface of the dhcp structure

In Suppl. Fig. 2 we show the calculated interatomic exchange of dhcp Pr, as a function of distance between atom pairs. In the left part of the figure, we illustrate how the exchange interactions of the surface atoms compare to those of the bulk dhcp Pr. The right part of the figure shows the corresponding data for the subsurface atoms. Note that both surface and sub-surface atoms have a distinctly different interatomic exchange compared to bulk values. For the top layer, the $\tilde{I}_{ij}$ are positive and ferromagnetic, moving closer to the antiferromagnetic exchange of the bulk as we move further away from the surface.

S3 Calculated magnetic specific heat

In Suppl. Fig. 3 we show the calculated magnetic contribution to the specific heat. Note that it exhibits no discontinuities that would indicate phase transitions; instead one notices only a Schottky anomaly peaked at about 30 K, apparently generated by the lowest-energy CF excited levels of the hexagonal site.

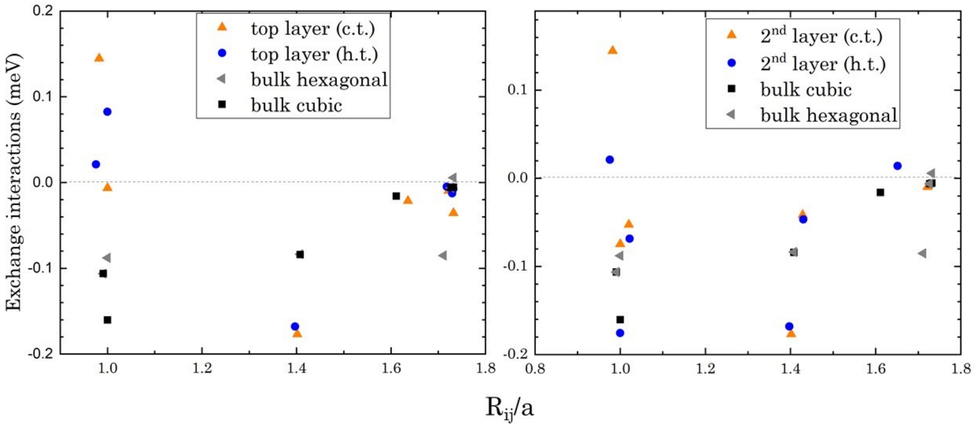

Supplementary Figure 2 (Color online) Exchange interactions $\tilde{I}_{ij}$ for the surface and the 2nd layer of Pr dhcp slab with hexagonal termination (h.t.) and cubic termination (c.t.).

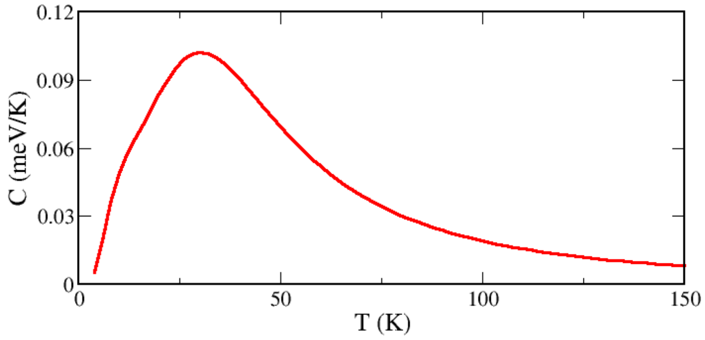

Supplementary Figure 3 Calculated magnetic contribution to the specific heat (per atom) of dhcp Pr.

S4 4*f* contribution to Kohn-Sham bands in dhcp Pr

In Suppl. Fig. 4 we show the "fat" Kohn-Sham band structure of dhcp Pr highlighting the Pr 4*f* character. The bands are calculated using the converged Kohn-Sham charge density obtained in self-consistent DFT+HI.

References

[1] Kamber, U., Bergman, A., Eich, A., Iuşan, D., Steinbrecher, M., Hauptmann, N., Nordström, L., Katsnelson, M.I., Wegner, D., Eriksson, O., Khajetoorians, A.A.: Self-induced spin glass state in elemental and crystalline neodymium. Science **368**(6494), 6757 (2020) https://doi.org/10.1126/science.aay6757

[2] Locht, I.L.M., Kvashnin, Y.O., Rodrigues, D.C.M., Pereiro, M., Bergman, A.,

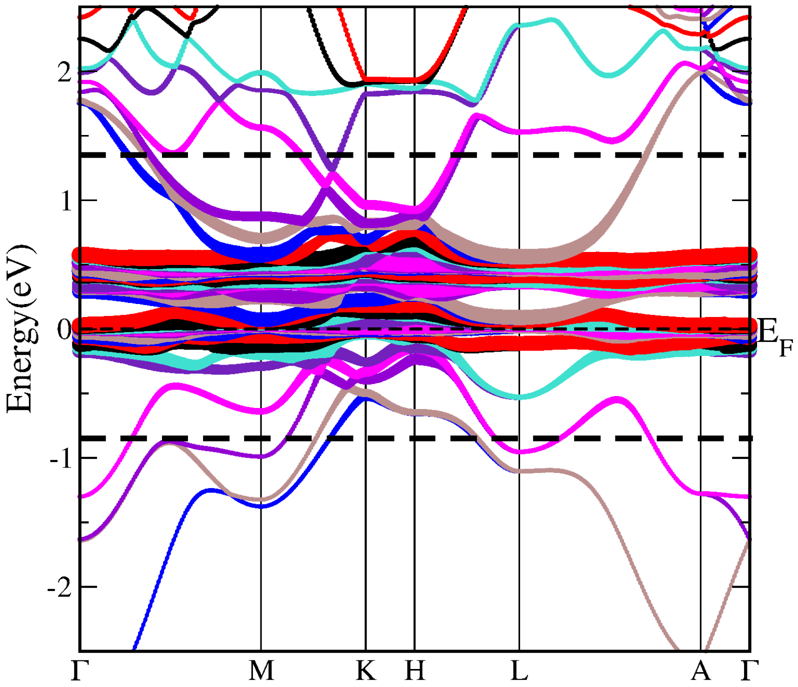

Supplementary Figure 4 Kohn-Sham band structure of dhcp Pr. The width of band dispersions indicates the magnitude of the $4f$ contribution. The boundaries of the window used in our calculations ([-1.1:1.1] eV relative to the centerweight of the $4f$ Kohn-Sham band) are shown by the bold dashed lines.

Bergqvist, L., Lichtenstein, A.I., Katsnelson, M.I., Delin, A., Klautau, A.B., Johansson, B., Di Marco, I., Eriksson, O.: Standard model of the rare earths analyzed from the Hubbard I approximation. Phys. Rev. B **94**, 085137 (2016) https://doi.org/10.1103/PhysRevB.94.085137
